# Teenagers as a Moving Target: How Can Teenagers Be Encouraged to Accept Treatment?

**DOI:** 10.3390/jpm2040277

**Published:** 2012-12-11

**Authors:** Pascale Gauthier, Jean-Michel Cardot

**Affiliations:** Biopharmaceutical Department, UFR Pharmacie, University d’Auvergne, 28, Place H. Dunant, B.P. 38, F-63001 Clermont-Ferrand, France; E-Mail: j-michel.cardot@udamail.fr

**Keywords:** pediatric drugs, teenagers, packaging, design

## Abstract

Pediatric patients exhibit their own needs and problems and are now considered as a real patient group in which downsizing the adult formulation is not the best choice and may result in problems. Adolescence (between 12 and 18 years) is a transitional period of life from puberty to adulthood and, in this pediatric subgroup population, complex problems are observed in compliance with chronic treatments. Heterogeneity exists in this group which follows very different and sometimes short trends and tendencies and where illness can be a problem leading to stigmatization. Influence of social environment as well as friends is complex in this period of life. Teenagers have to take care of themselves and be part of the treatment including all the features of the social code of this group. Particular attention has to be paid to formulation and packaging in order to increase compliance and to suit the specific needs of this pediatric subgroup. Some examples are given for different drug forms.

## 1. Introduction

Medical and pharmaceutical industries have seen a significant evolution in the past century. We have moved from medicinal products prepared specially by the pharmacist following a doctor’s prescription, to industrial forms not always adapted to patients’ specific needs [1]. This homogenization of the formulation and presentation of medicine allows not only greater certitude of the doses administered, but also an increase in constancy of the quality, in productivity, and reproducibility. It also diminishes the risk of decrease in compliance due to inaccurate preparation. Nowadays, we observe that the adjustment of the medications (and of the prescription) for each patient is at the center of health strategies for particular population groups. This paper focuses on a specific subgroup of the child population, namely teenagers. It will investigate who they really are as patients, how we can observe problems of compliance in this group, what we can do to improve treatments, and select drugs adapted to their needs. The pharmaceutical industry must understand that teenagers are the adults of tomorrow and this represents a huge challenge for the future of our medicine. 

## 2. Presentation of Problems Specific to Teenagers

**Figure 1:**
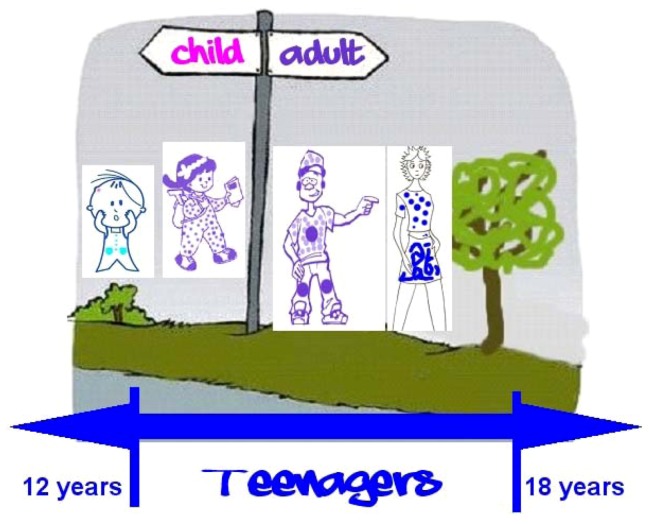
Teenagers and age scale.

From a legal point of view the teenagers remain in the group of children. All the definitions present the teenager as an adolescent, a teen, a juvenile person not fully developed and more generally, depending on the country (Figure 1) [1,2,3], as a person male or female between 12 and 18 years. Adolescence can also be seen as a “middle time” between childhood and adulthood and is a transitional period of life which, from puberty to adulthood, is characterized by marked physiological changes. It can be viewed as a transitional state, a time to define oneself with development of identity and of sexual feelings.

We must bear in mind that teenagers are undergoing a transformation. They are searching for their own identity, and trying new things. They represent a heterogeneous and moving group, following different and sometimes very short-lived tendencies. For marketing, teenagers can be seen as a huge challenge and a real opportunity. Clearly, the market needs to understand these consumers that yesterday were “kids,” are now “teenagers,” and tomorrow will be “adults” [4].

Some authors describe marketing to the young in different age groups with different tendencies [5,6]. Thus, while teenagers still remain a mystery for adults, who are always scrambling to find ways to understand and to live with them, they are carefully studied. We find them as the main dedicated topic of newspapers and internet blogs. In publications, a number of topics often appear, such as: “teenagers and their problems of identity”; “teenagers: a mutation?”; “teenagers and different times: 11–13 years: no longer kids; 13–15 years: friends come first; 15–18 years: individual tastes are revealed.” These underline the complexity and the changes occurring in these age groups. 

Specific problems exist such as:

- Relations between girls and boys: are they at war or simply living in separate worlds?
- Problems with nutrition (junk food) in the form of overweight/obesity/bulimia/anorexia. Possible addiction to alcohol or drugs;
- Strong need to stay connected and possible addiction to the mobile phone, the internet and also a great ability in understanding the numeric world;
- Out-of-bounds behavior and sensation of being unbreakable.

Teenagers can be seen to represent a world apart, but are, at the same time, the adults of tomorrow that inspire trends and innovations even if sometime they remain as young people who want to be loved by their parents.

Youth marketing is a reality studied and described by different economists [5]; where we find different age groups, three of them could be linked to their behavior:

- 11–14 years: a moving target with infantile desires and aspirations of adulthood;
- 14–18 years: corresponding to the research of pure marketing, where the internet is the preferred medium and products are carefully selected;
- a last group, comprising young adults between 18 and 24–30 years, are no longer teenagers but are still driven by similar passions where underground marketing is used with a lot of instability.

This last group emphasizes the fact that teenager behavior can last beyond 18 years and may last from 15 to 30 years depending more or less on the individuals and their way of life, family and financial circumstances. No clear rules can be established, so this target can be considered for a longer period than up to 18 years and can include the target of younger adults that want to stay “teenagers”. 

Teenagers’ comportment corresponds also to contrasting attitudes. They can be either shy and have difficulties in communication, or at the opposite end of the scale, can appear very sure of themselves. They know everything better than adults (especially their parents) and they do not want any help; they are sometimes arrogant and feel “that they are invincible.” In this case, it is difficult for them to understand disease, a perception which may be closely linked to induced disabilities. For example, acne can be seen for them as a severe disease that modifies physical appearance. In such a case, a treatment with side effects and constraints can be acceptable to teenagers. On the other hand, as another example, in the area of contraception, where it is always difficult to explain and to be well understood and where, as a consequence, there is an increased number of undesired pregnancies and increasing use of the “morning after” pill as regular contraception.

## 3. How to Adapt Treatments to Teenagers Needs

According to the problems and tendencies previously described, which products can be suggested for better use of medicine by teenagers?

Improving the use of medicine first requires an understanding of the relationship between teenagers and their disease and the type of disease. Chronic or acute disease can induce different behavior in young patients according to age. Table 1 summarizes the description of the European Medicines Agency (EMA), where differences between teenagers and children are highlighted.

**Table 1:** Behavior of patient as a function of the type of disease [7].

TYPE OF DISEASE	PATIENTS	
	CHILDREN	TEENAGERS
**ACUTE**	**Children unwell:** fever/pain less cooperative	Teenager ill and unwell
**DISEASE**	**Liquid forms often preferred**	Tablets, injectables if needed **easiest and more rapid form**
	In case of vomiting rectal or injection necessary	
**CHRONIC DISEASE**	Sometimes from early age, for chronic disease continuous treatment	Reciprocal impact of chronic disease in adolescence
**LONG TERM DISEASE**	Preferable to **offer large range of dosage forms** to allow choice to caregivers	**Problems of compliance** with all treatments
	In case of regular injections needed to reduce pain	

Compliance is described as the degree to which a patient correctly follows medical advice. For chronic diseases, the World Health Organization indicates that 50% of patients do not comply. Compliance decreases with the children’s age and is shown for one month treatment to be 82% for 7–9 years, and 71.6% for 10–16 years. This can be related to the chronic disease itself (long term), the administration time and regimen, side effects or non adapted dosage form [4,8]. 

In its guidelines, EMA gives matrix tables of dosage form related to age [7]. These proposals emphasize the fact that teenagers are fully actors in their treatment. 

**Figure 2:**
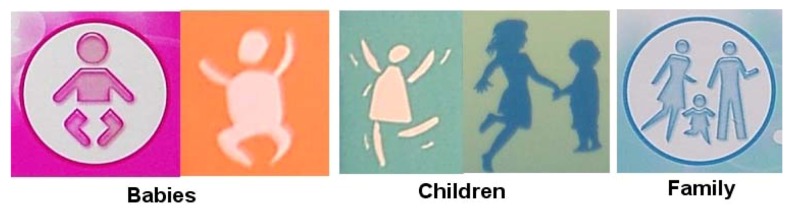
Pictograms showing users.

**Figure 3:**
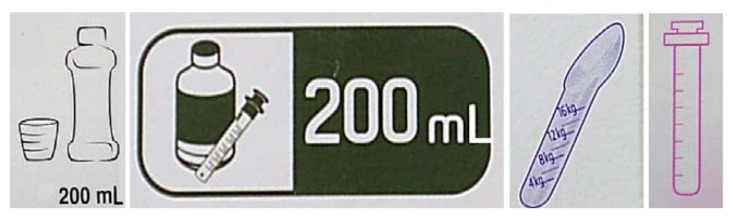
Pictograms showing products.

**Figure 4:**
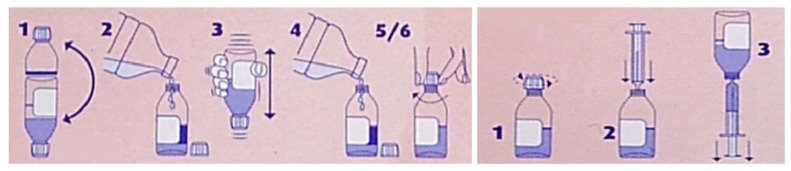
Pictograms explaining use of medicinal products.

Packaging is the first element of medicinal treatment that appears to be relevant for the teenagers. A drug packaging—as with all packaging—is required to perform two functions: containment and protection [9,10]. Many pictograms are now available on packaging and these can help to identify the user age group (Figure 2) though there is as of yet none for teenagers. Pictograms can also underlined product characteristics (Figure 3) or indicate explanations of use of medicinal products (Figure 4).

Materials are becoming more efficient and can be seen as real partners in improvement of treatment through design that can be defined as the concept of creating an object that is of value which must be functional in all areas of everyday life [10,11,12]. Integration of design in pharmacy can lead to drugs that offer better adaptation to user’s requests. This is an absolute requirement for the target of teenagers where we need to understand and follow their needs.

**Figure 5:**
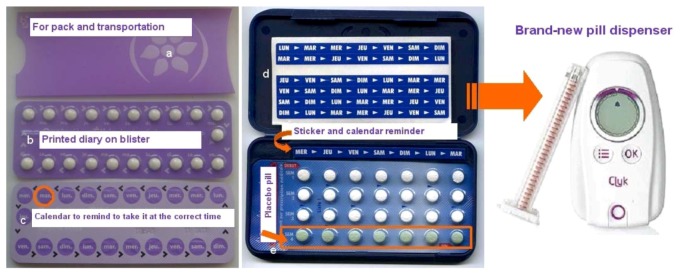
Examples of pills blisters [13].

For all type of drug dosage form, understanding and monitoring treatment is fundamental. For a chronic treatment, compliance is essential and in some cases the packaging can help as a reminder of the treatment. Looking to peroral forms, they are shown as the “form of choice” for teenagers [7]. Tablets and capsules are small in size, easy to take and to transport when they want to be taken discreetly. In the specific case of orodispersible tablets, they can be taken without water and their flash dissolution avoids problems of deglutition. For tablets with modified release, they can be taken once a day, for example, in the morning before high school, thus avoiding stigmatization. At any time, these forms must be taken correctly (for example, avoiding crushing the coated tablets in case of coated modified release forms). The methods to improve compliance were first initiated for contraceptive drugs. Contraceptive treatments often begin in the teenager age group and compliance is important to avoid unwanted pregnancies. Much has been done for pills with slim blister packs for transportation (Figure 5a) with, for example, a diary printed directly on the blister (Figure 5b,c) or stickers (Figure 5d) that underline the time to take pill and help follow regularly treatment. In some cases, there are also placebo tablets (Figure 5e) that avoid any treatment interruption. These techniques are used nowadays for other chronic treatments and especially the use of packaging devices which include reminder systems improving adherence and reducing catch oversights. Due to progress in materials, we can find ever-slimmer packaging which is easier to transport. Currently, products are appearing which are using new technologies as reminders to take drugs (SMS, smartphone applications) which are helpful for all chronic treatments, and are also present in the targeting of contraceptive pills. As we have remarked previously, teenagers are very fond of new technology and high-tech gadgets. Just recently (2012-10-09), the Clyk™ has appeared on the market. It is an intelligent tablet dispenser co-developed by Bayer Medical GmbH and Balda Medical GmbH and rewarded with a prize for intuitiveness and ease of use. It is particularly useful in the area of the contraceptive pill that always appears as an innovative target and allows management of the treatment as well as dispensing pills [13]. 

Special formulations could be developed for teenagers. However those formulations must not be confused with candies and other foods. As an example, chewable tablets, appreciated by children and teenagers, are used for headaches or colds in U.S., but not allowed in France. Other new forms such as buccal strips can be used successfully by teenagers. These buccal patches which deliver compounds with good permeation via the buccal mucosa are well accepted and used for this target. They avoid the “first pass through liver effect” and increase the oral bioavailability of the drugs. They need lower doses for similar therapeutic effects (with fewer or reduced side effects). As less active pharmaceutical ingredient (API) is employed, cost is decreased. They can be presented as new attractive designs for safer pain-free oral delivery, which is also ideal for all patients who present difficulty in swallowing.

**Figure 6:**
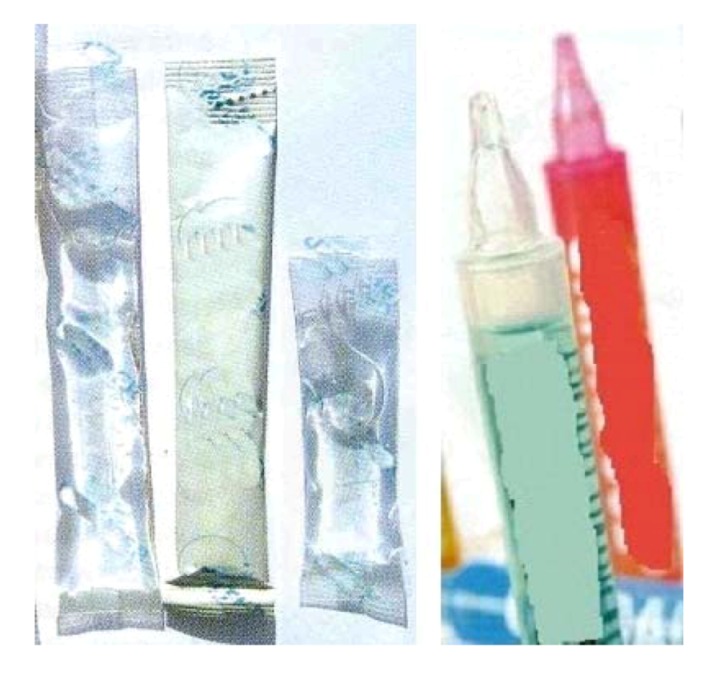
Examples of single-dose systems for oral liquid drugs [16,17].

Other oral forms are represented by oral liquid form [1,10,14,15]. These forms can also have preferred acceptability by the teenagers as well as being easy to take without water. Single-dose systems such as sticks (Figure 6) or single-dose products are clearly helpful for a nomadic and discreet use specially required for the target of teenagers. 

These smart products can help them take their medicine easily, anywhere they want, and construe drugs as “fun products” that can be presented to their friends while avoiding stigmatization. However, at any moment for liquid drugs, we must take care of the flavor and avoid bad taste. The serious problem of overconsumption is less important in the case of teenagers, because they can understand and master the treatment, thus resulting in fewer unintended errors than for the youngest children where we also need to offer attractive systems to improve compliance.

**Figure 7:**
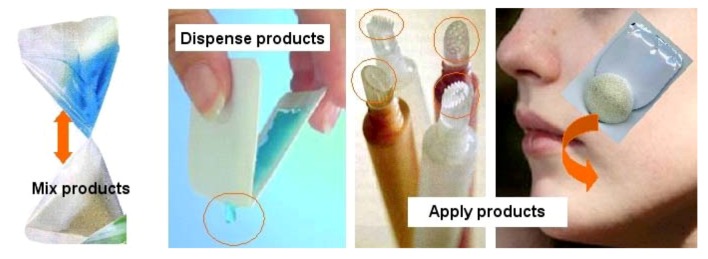
Example of single-dose dispensing systems for cream and gels [18,19,20,21].

In the case of acne, in addition to peroral formulations, topical forms such as creams and gels are commonly found. Easy to use and apply, products that present a fresh and cosmetic-like formulation are considered as a must. As acne can be very badly accepted by teenagers where physical appearance is important, formulations need to produce a rapid result. Even for boys, the use of “cosmetic-like” products is now an essential part of wellbeing and becoming the usual form of treatment. Specific products exist which highlight skin treatment and include the use of cosmetics to give the impression of being fresher after an overnight party. Products for acne can be presented as a single-dose system that may integrate an application and/or dosing system. All single use pre-dosing systems (Figure 7) are very well accepted due to their small size and ease of transportation. Some systems produce foam, offering an innovative aspect for drugs which appear much more important for the teenager as all new products enhanced by an innovative packaging. Most of these systems are now taking care of sustainability with a decrease in piece numbers for manufacture and are using recyclable materials, which is also important for acceptability by teenagers.

**Figure 8:**
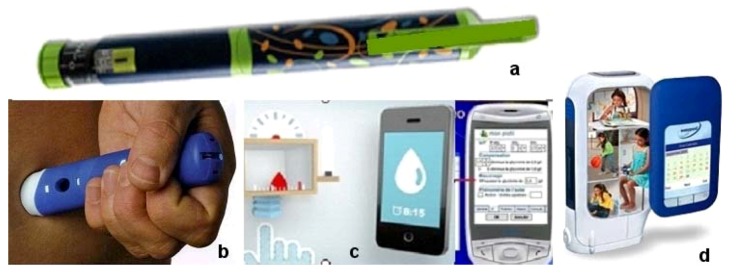
Example for injectable forms [25,26].

Other chronic treatments for some severe diseases use regular injections. In this case, it is necessary to reduce or limit pain. For example, type I diabetes can start at puberty and regular injections, as well as regular blood glucose testing, are necessary. This severe chronic disease can be badly accepted by this young population [22,23]. An important challenge is to be able to offer them possibilities of new systems to integrate the injection of insulin in regular life. Simple and rapid devices that avoid stigmatization and offer a small size with intuitive use are available with injection pens that can be customized (Figure 8a) and allow, at the same time, very small and precise dosage of drugs to be delivered. The choice of the customized system can be really important for increasing compliance in the young. Such devices can, by the use of new technologies, provide reminder times for taking the drugs and incorporate mobile phone messages. Some add-on equipment such as glucometers can now be linked with mobile smartphones under various operating systems [24]. These are helpful in monitoring and calculating insulin dosage according to food intake, sport and other parameters and archiving all the results which can be transferred directly to the medical device (Figure 8c). In addition to the important role in monitoring, these systems are interesting for teenagers as they can be considered as nomadic connected systems close to a social network communication type. As a final example, a new device is being produced by Merck Serono for targeting growth hormone treatment. Easypod^®^ (Figure 8d) is an automated drug delivery system that allows a complete management of treatment with dosage administration including a reminder of the precise time of administration. All this is done to help the monitoring and good compliance in young patients where the full device is completely conceived as a partner in the therapy [25].

Of these three examples, the problems highlighted in table I have been studied, and possible solutions have been put forward. More examples following these ideas can be found for many other galenical forms.

## 4. Conclusions

This overview shows that teenagers can be seen as a vast, but also moving target, difficult to define according to the strict age limits of the definition. Adolescence is really the time of metamorphosis where all is changing and the teenager is “between two worlds” (children and adults). In the case of chronic disease during this period, new information has to be incorporated as parents’ influence is decreasing or even perceived as negative. Most of the time, priorities are moving for teenagers according to their environments (friends, rejection of parents’ authority) and any treatment is clearly an obstacle that can be badly accepted by those, who more often than not, want to be accepted by new groups of friends. Advice may be rejected, leading sometimes to a disease being dissimulated with the consequence that compliance of treatments appears as a huge problem! Adaptation of formulation and treatment for teenagers is particularly fundamental and represents a challenge not only for the pharmaceutical industry but also for health systems. The target of the teenager is socially well studied but sometimes unknown and quite new for the pharmaceutical industry that must understand that it is fundamental to produce drugs adapted to their needs which must include their requests and avoid any stigmatization. Dosage forms as well as devices or packaging systems must help them to integrate the treatment into everyday life easily. At the end of the day, industrials thinking of teenagers as specific targets must also think of them as future adults, and the long-term consumers that they will eventually become.
